# Absence of Curli in Soil-Persistent *Escherichia coli* Is Mediated by a C-di-GMP Signaling Defect and Suggests Evidence of Biofilm-Independent Niche Specialization

**DOI:** 10.3389/fmicb.2018.01340

**Published:** 2018-06-22

**Authors:** Yinka M. Somorin, Tara Vollmerhausen, Nicholas Waters, Leighton Pritchard, Florence Abram, Fiona Brennan, Conor O’Byrne

**Affiliations:** ^1^Discipline of Microbiology, School of Natural Sciences, College of Science, National University of Ireland, Galway, Ireland; ^2^The James Hutton Institute, Dundee, United Kingdom; ^3^Soil and Environmental Microbiology, Teagasc, Johnstown Castle, Ireland

**Keywords:** curli, biofilm, soil, c-di-GMP, RpoS, *Escherichia coli*

## Abstract

*Escherichia coli* is commonly viewed as a gastrointestinal commensal or pathogen although an increasing body of evidence suggests that it can persist in non-host environments as well. Curli are a major component of biofilm in many enteric bacteria including *E. coli* and are important for adherence to different biotic and abiotic surfaces. In this study we investigated curli production in a unique collection of soil-persistent *E. coli* isolates and examined the role of curli formation in environmental persistence. Although most soil-persistent *E. coli* were curli-positive, 10% of isolates were curli-negative (17 out of 170). Curli-producing *E. coli* (COB583, COB585, and BW25113) displayed significantly more attachment to quartz sand than the curli-negative strains. Long-term soil survival experiments indicated that curli production was not required for long-term survival in live soil (over 110 days), as a curli-negative mutant BW25113Δ*csgB* had similar survival compared to wild type BW25113. Mutations in two genes associated with c-di-GMP metabolism, *dgcE* and *pdeR*, correlated with loss of curli in eight soil-persistent strains, although this did not significantly impair their survival in soil compared to curli-positive strains. Overall, the data indicate that curli-deficient and biofilm-defective strains, that also have a defect in attachment to quartz sand, are able to reside in soil for long periods of time thus pointing to the possibility that niches may exist in the soil that can support long-term survival independently of biofilm formation.

## Introduction

*Escherichia coli* is commonly associated with the gastrointestinal tract of humans, warm-blooded animals, and reptiles (Berg, 1996; Gordon and Cowling, 2003), and its presence in the external environment is often used as an indication of recent fecal contamination. This niche specificity underpins its use as an indicator of fecal contamination in the environment. Nevertheless, *E. coli* has been isolated from various sources outside of its primary habitat (Ishii et al., 2006; Chiang et al., 2011; Byappanahalli et al., 2012) and it persists and grows in external environments such as subtropical waters and sediments (Anderson et al., 2005). In fact, some *E. coli* linages have been reported to exhibit primarily a non-host lifestyle (Walk et al., 2009). Brennan et al. (2010) reported that *E. coli* are capable of long-term colonization and persistence in an lysimeters that had not been exposed to fecal material during a 10-year period prior to their isolation. These soil-persistent *E. coli* strains are genetically diverse and possess unique growth and metabolic characteristics that suggest adaptation to soil conditions (Brennan et al., 2013). When *E. coli* enters the soil, there is rapid decline in the population, but a part of the population is able to persist due to inherent physiological properties or has an ability to colonize favorable niches in the environment (Ogden et al., 2001).

While it has been shown that the general stress response regulator, RpoS, is important for long-term persistence of *E. coli* in soil (Somorin et al., 2016), the exact mechanisms for their survival in the soil environment remain unclear. Some genetic factors are known to enhance bacterial survival in the different environments. For example, flagellin was identified to help *Pseudomonas aeruginosa* to adhere to soil amoeba and persist in soil (DeFlaun et al., 1990). A functional flagellum was shown to be important for attachment and colonization of infant mouse bowels by *Vibrio cholerae* (Attridge and Rowley, 1983). Exopolysaccharides and type 1 aggregative adherence fimbriae were found to support *in vivo* colonization of germ-free mice and biofilm formation in *E. coli* O104:H4 (Al Safadi et al., 2012). More recently, Yad fimbriae were demonstrated to promote *E. coli* adherence to plants, animal cells and promote persistence in the environment (Larsonneur et al., 2016). Production of biofilm enhances the survival of *Salmonella* in a dry and nutrient-depleted environment (Vestby et al., 2009) and of *E. coli* in soil (Truhlar et al., 2015). Biofilm has also been shown to promote the persistence of *E. coli* on fresh produce (Annous et al., 2009) and in food processing environments (Silagyi et al., 2009; Maal-Bared et al., 2013). Curli form a major component of biofilm in many enteric bacteria including *E. coli* (Barnhart and Chapman, 2006; Yaron and Römling, 2014). Curli are crucial for adherence to plant and animal tissues, plastic and stainless steel by *E. coli* and Salmonella (Patel et al., 2011; Fink et al., 2012; Yaron and Römling, 2014; Carter et al., 2016). Although curli are important for attachment of *E. coli* to biotic and abiotic surfaces, little is known about their contribution to persistence in a soil environment. Brombacher et al. (2003) previously reported that presence of curli enhanced retention of *E. coli* in sand columns, however, curli production in *Salmonella* spp. did not have an impact on their retention in sand (Salvucci et al., 2009).

Since biofilm formation is thought to play an important role in the survival of *E. coli* in the environment (Vogeleer et al., 2014), it was hypothesized that environmentally adapted *E. coli* would retain the capacity to produce biofilms. However, three out of five soil-persistent *E. coli* strains in our previous study were unable to produce biofilms in microtiter plates (Somorin et al., 2016). This raises questions about the ability of these soil-persistent *E. coli* to produce the extracellular matrices (ECM) that make up biofilm. Curli fimbriae are proteinaceous fibers which consist of over 85% by mass of the ECM produced by *E. coli* (McCrate et al., 2013), but it is unknown whether they are important for long-term soil persistence. This study investigated a unique collection of phylogenetically diverse, long-term soil-persistent *E. coli* isolates to investigate the prevalence of curli-negative strains and understand the role of curli and attachment in soil persistence. A significant subset of soil-persistent strains were found not to produce curli and the basis for this phenotype was investigated further. Some of these curli-deficient strains were found to carry mutations in genes involved in c-di-GMP metabolism, which are known to influence curli expression (Sommerfeldt et al., 2009; Lindenberg et al., 2013). This present study shows that curli are important for attachment of *E. coli* to sand but are dispensable for soil survival, and suggests that *E. coli* may occupy niches within the soil environment that does not rely on biofilm formation.

## Materials and Methods

### Bacterial Strains Used and Growth Conditions

Long-term soil-persistent *E. coli* strains, which were isolated from leachates obtained from lysimeter units (Brennan et al., 2010) and belonging to distinct phylogenetic groups were used in this study (Supplementary Table S1). Some of these strains have been previously described (Brennan et al., 2010; Somorin et al., 2016). Two commensal strains (SE11 and SE15) and a well-studied laboratory strain (BW25113) were used for comparative purposes. The mutant strains from the BW25113 background were obtained from the Coli Genetic Stock Centre (Yale, United States) and the kanamycin resistance cassette used in constructing the mutants was removed by FLP-FRT recombination, and removal of the cassette was confirmed by plating on Luria-Bertani (LB) agar with 50 μg ml^-1^ kanamycin (LBKan).

The PdeR^E620K^ point mutation was created in BW25113 using homologous recombination by λ-red-recombinase using pKOBEGA with a single-stranded DNA oligonucleotide (5′-AATCTTCAGGTGATCGCCGAAGGCGTCAAAAGCGCCAAGGAAGATGCTTTTTTAACCAAG-3′) (Costantino and Court, 2003). An additional 13 bp silent mutations were introduced to enable detection of recombinants by PCR. PdeR^E620K^ mutations were selected based on color morphology after being grown on CR-YESCA at 28°C for 48 h. The introduction of the PdeR^E620K^ point mutation was confirmed by sequencing the *pdeR* PCR product (using primers: Forward, 5′-TTATGCGCGCTTCAGATAG-3′; Reverse, 5′-CATAAACCTGCGAGTGGCG-3′).

### Congo Red Assay

Curli production was determined in the strains by Congo Red assay as previously described (Zhou et al., 2013). Congo Red agar plates was made by preparing yeast extract and Casamino acid agar (YESCA; 1 g L^-1^ yeast extract, 10 g L^-1^ casamino acids, 20 g L^-1^ agar) and after autoclaving, filter sterilized Congo Red (50 μg ml^-1^ final concentration; Sigma) and filter sterilized Brilliant Blue G (10 μg ml^-1^ final concentration; Sigma) were added. *E. coli* strains were grown in LB broth and incubated at 37°C overnight. Five microliters of the overnight culture of each strain was spotted on the center of a thick Congo Red agar plate. The plates were incubated at 28°C for 48 h. Images were captured with Canon CanoScan 9000F MKII Flatbed Scanner at 600 dpi.

### Western Blot for CsgA, RpoS, and CsgD

Protein extraction and western blot analysis was used to analyze bacteria-associated curli as previously described (Zhou et al., 2013). For CsgA, whole-cell samples from YESCA plates were resuspended in 1 ml of Potassium phosphate (KPi) buffer and normalized to OD_600nm_ of 1.0. Each pellet resulting from 150 μl of normalized cell suspension was resuspended in 70 μl of 100% Hexafluoroisopropanol (HFIP) to dissociate the curli subunits and the HFIP was removed by vacuum concentration (Concentrator plus; Eppendorf) at 45°C for 30 min. The dried cell pellets were resuspended in SDS-PAGE loading buffer and boiled at 95°C for 10 min. An equal amount of protein from each sample was separated using a 15% SDS–PAGE gels at 100 V for 1 h. After electrophoresis, proteins were blotted onto a polyvinylidene difluoride (PVDF) membrane using a semidry system (Jencons, United Kingdom) at 3 V for 1 h. A blocking step with 5% (w/v) skim milk in Tris-buffered saline with 0.05% Tween 20 (TBST) was performed, and the membrane was incubated in 5,000-fold diluted anti-CsgA antibody (gift from Matt Chapman) in the blocking solution. Blots were washed in TBST three times for 10 min each and incubated in 10,000-fold diluted goat anti-rabbit IgG conjugated with horseradish peroxidase (Santa Cruz). After washing three times in TBST, the blot was developed with Amersham ECL Prime Western Blotting Detection (GE Healthcare), prior to exposure to photographic film and CsgA bands observed following development of the film in a Kodak X-ray developer. CsgD and RpoS were detected using a similar approach except that there was no HFIP treatment involved. CsgD was detected using anti-CsgD antibody (gift from Shinya Sugimoto) diluted 2,000-fold in SignalBoost Immunoreaction Enhancer Solution 1 (Merck Millipore) and anti-rabbit IgG HRP (Santa Cruz) diluted 20,000-fold in SignalBoost Immunoreaction Enhancer Solution 2 (Merck Millipore). RpoS was resolved on 10% gel and detected by 5,000-fold diluted mouse monoclonal anti-RpoS antibody (Santa Cruz) and 3,000-fold diluted anti-mouse IgG HRP (Santa Cruz).

### Bioinformatics

All nucleotide sequences for *E. coli* K-12 MG1655 were obtained from the EcoCyc *E. coli* database^1^. Nucleotide sequences of *E. coli* SE11, SE15, BW25113, and W3110 were retrieved from the National Centre for Biotechnology Information (NCBI) database with GenBank Accession numbers AP009240.1, AP009378.1, CP009273.1, and AP009048.1, respectively. Unassembled genomic sequences of the soil-persistent *E. coli* were provided by F. P. Brennan (unpublished data). Nucleotide sequences of the genes of interest were extracted from the contigs of the soil-persistent strains using Geneious R8 (Biomatters), the nucleotide sequences were translated to amino acid sequences and multiple sequence alignment was performed using Clustal Omega (Sievers et al., 2011). For the graphical representation of the sequence alignments, ClustalW in BioEdit v7.2.5 (Ibis Bioscience, United States) was used. The phylogenetic analysis was performed using kSNP3.0 (Gardner et al., 2015) to construct an alignment-free SNP-based phylogeny from the eight curli negative strains (Sequence data available at BioProject Accession # PRJNA420620) and representatives from all of the Clermont 2013 phylogroups (Clermont et al., 2013), using a *k* value of 31. The accession numbers for these strains can be found in Supplementary Table S2. The resulting parsimony tree was visualized with FigTree v1.4.3 and annotated using Inkscape.

### Overexpression of PdeR, DgcE, and Curli Expression

The curli-positive and curli-negative soil strains were transformed with pCAB18 plasmid (IPTG-inducible low copy number vector carrying the *P*tac promoter, Amp^R^) carrying wild type *pdeR* (pCAB18-*pdeR*; gift from Regine Hengge). The transformed strains were grown on Congo Red-containing Yeast Extract and Casamino acid agar (CR-YESCA) with IPTG (10 μM) and ampicillin (100 μg ml^-1^) at 28°C for 48 h to induce overexpression of PdeR. CsgD protein expression was determined in the strains overexpressing PdeR grown on YESCA, as described above. Wild type DgcE from *E. coli* BW25113 was cloned into TOPO-XL-PCR (Invitrogen). Transformed curli-negative soil strains with pCR-XL-TOPO-*dgcE* were grown on CR-YESCA at 28°C for 48 h.

### Soil Survival Assay

Survival of *E. coli* BW25113 and its corresponding Δ*rpoS*, Δ*csgA*, Δ*csgB*, and Δ*csgD* mutants in silty loam soil was determined as described by Somorin et al. (2016). Silty loam soil, which had no detectable background levels of *E. coli*, was sieved with a 2-mm sieve and 1 g weighed into a series of 15 ml tubes. Equal cell numbers were first washed in PBS and then inoculated into separate soil samples (to give 1 × 10^7^ CFU g^-1^ of soil), inverted 10 times by hand, slightly capped to allow air exchange, and incubated at 15°C. As a control, 50 μl of sterile PBS was added to 1 g of silty loam soil. The experiment was set up in triplicate. Inoculated soils were destructively sampled at different time intervals to determine the survival of the wild type and mutant strains. For cell recovery, 2 ml of PBS was added to each tube containing soil sample, capped and mixed by inverting the tube three times and vortexed for 2 × 20 s. The resulting soil slurry was allowed to settle for 2 min and 20 μl of the supernatant liquid was collected and serially diluted. Ten microliters of all dilutions were plated in triplicate on MacConkey agar (Sigma) and incubated at 37°C overnight. Colonies were counted to enumerate the viable cells at each time-point.

### Sand Attachment Assay

Sand attachment assay was conducted according to the method described by Hinsa et al. (2003). *E. coli* cells were grown for 24 h on LB agar at 28°C. Colonies were scraped off with a loop and resuspended in 5 ml PBS. OD_600nm_ was measured and normalized in LB without salt (LBns) to give a starting population of 10^6^ CFU ml^-1^. Quartz sand was pre-weighed into 1.5 ml tubes and sterilized by autoclaving. Then, 0.5 g of sterilized quartz sand (Sigma) was added to wells of a 96-well plate and 1 ml of the inoculated LBns was added to each well. Plates were incubated static in the dark at 28°C for 48 h. After incubation, the LBns in each well was removed, serially diluted, and plated out to determine planktonic cell count. For biofilm cell count, sand in the 96-well plate was pipetted into pre-weighed 1.5 ml tubes and 500 μl of PBS was used to wash the sand five times to remove unattached cells. All liquid was removed and tubes were re-weighed. Then 500 μl of PBS was added and the tubes were vortexed for 30 s, sonicated (4 min, 100% power) and vortexed again for 30 s. The liquid fraction was then serially diluted and plated onto LB agar to determine the biofilm count. The bacterial cell counts were normalized to the weight of the sand.

### Statistical Analysis

The Student’s *t*-test was used when comparing means from two samples whereas one-way Analysis of variance (ANOVA) was used when comparing means from three or more samples. Statistical comparisons among the means in ANOVA were compared using Duncan Multiple Range Test at 5% probability level. Error bars on graphs indicate standard deviations from the means. Differences in soil survival and sand attachment were investigated using GraphPad Prism 6.

## Results

### Curli Production Varies Among Soil-Persistent *E. coli*

**FIGURE 1:**
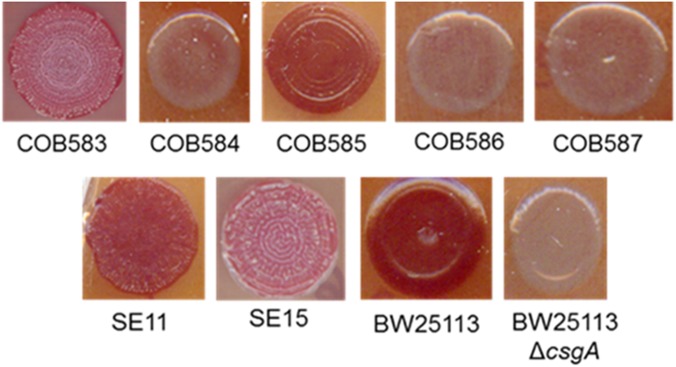
Curli production among soil-persistent *Escherichia coli*. Macrocolonies of soil-persistent, commensal and control strains were grown on Congo Red-containing Yeast Extract and Casamino acid (CR-YESCA) agar at 28°C for 48 h. *E. coli* BW25113 was used as the positive control and *E. coli* BW25113 Δ*csgA* was the negative control.

Curli production was determined in five soil persistent strains using a Congo Red (CR) assay as previously described (Zhou et al., 2013). CR binding among soil-persistent *E. coli* revealed the formation of red colonies on CR-YESCA for COB583 and COB585 whereas COB584, COB586, and COB587 formed white or pale pink colonies (**Figure 1**). Human commensal *E. coli* isolates, SE11 and SE15, were used for comparison purposes and both stained red on CR-YESCA agar indicating curli production. *E. coli* BW25113, which was used as the positive control, was red while the negative control, *E. coli* BW25113Δ*csgA*, was white, confirming that the red staining was dependent on curli production. These data suggested that three of the soil strains (COB584, COB586, and COB587) were not expressing curli under these growth conditions, a finding that is consistent with the inability of these strains to produce biofilm (Somorin et al., 2016). The morphology of the macrocolonies in the curli-positive strains varied from red, dry and rough (rdar) morphotype (as in COB583, SE11, SE15) which suggests the cells produced curli and cellulose (Bokranz et al., 2005) to brown-red, dry with large rings (as in COB585 and BW25113) (**Figure 1**), which suggests the macrocolonies produced curli only (Bokranz et al., 2005). Preliminary screening of COB584, COB586, and COB587 for cellulose production on agar plates containing Calcoflour white, showed that COB584 and COB587 produced cellulose but not COB586 (data not shown). When the total collection of 170 soil-persistent *E. coli* isolates was analyzed, 153 (90%) were curli-positive and 17 (10%) were curli-negative (Supplementary Table S3).

### Curli Transcriptional Regulator CsgD and Curli Major Subunit CsgA Are Not Expressed in Curli-Negative Soil-Persistent *E. coli*

**FIGURE 2:**
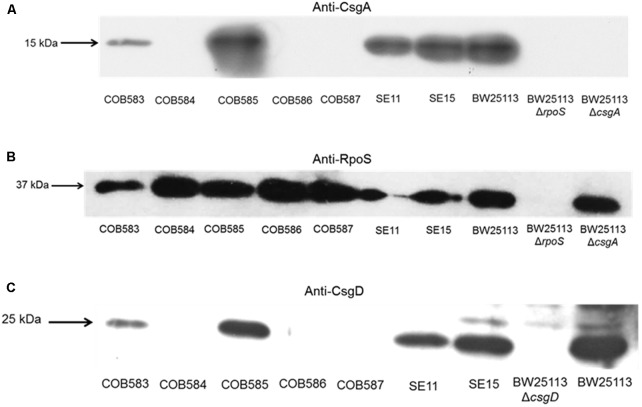
CsgA and CsgD are not expressed in curli-negative soil-persistent *Escherichia coli* strains. Cells of soil-persistent, commensal and control strains were grown on Yeast Extract and Casamino acid (YESCA) agar at 28°C for 48 h. Protein extraction and western blotting was done for determining the expression of CsgA **(A)**, RpoS **(B)**, and CsgD **(C)**.

To understand the reason for the loss of curli production in the three soil-persistent *E. coli* strains (COB584, COB586, and COB587) unable to produce biofilm (Somorin et al., 2016), the expression of the major curli subunit CsgA, which is essential for curli production, was investigated. Western blotting analysis showed that these three curli-negative strains did not express CsgA, whereas the curli-positive strains COB583 and COB585 did (**Figure 2A**). Since the expression of curli is regulated by RpoS, the ability of the curli-negative strains to express RpoS under the curli-inducing condition (YESCA agar at 28°C for 48 h) was investigated. All the curli-positive and curli-negative strains expressed RpoS under this condition (**Figure 2B**). Since RpoS regulation of curli expression occurs through the curli transcriptional regulator CsgD, which activates the transcription of *csgBAC* operon, the ability of the strains to express CsgD on YESCA agar was determined. CsgD was also not expressed in the curli-negative *E. coli* COB584, COB586, and COB587 but was expressed in all the curli-positive strains (**Figure 2C**). This suggests that the loss of curli in these strains was caused by the absence of CsgD and suggests some defect in regulation upstream of CsgD in the curli production regulatory pathway.

### Sequence Analyses Reveal That Mutations in the GGDEF Domain of DgcE and in the EAL Domain of PdeR Are Associated With the Curli-Negative Phenotype

**FIGURE 3:**
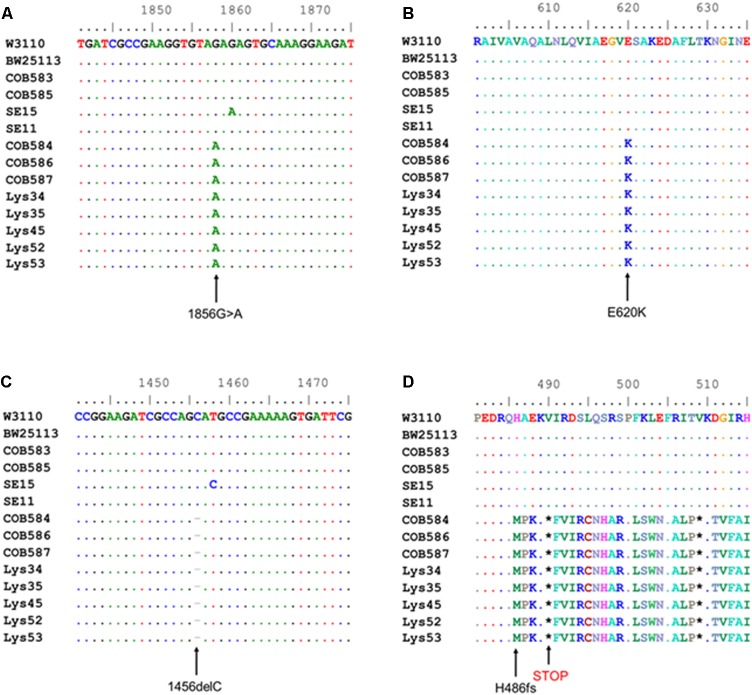
Multiple sequence alignment shows mutations in cyclic-di-GMP metabolism genes in curli-negative soil-persistent *Escherichia coli*. Multiple sequence alignment of nucleotide sequences in cyclic di-GMP phosphodiesterase-encoding gene (*pdeR*) among 170 soil-persistent strains in our collection shows a point mutation (1858G > A) **(A)** which led to an amino acid change in the PdeR protein sequence (E620K) in COB584, COB586 and COB587 and five additional soil-persistent strains **(B)**. Alignment of the nucleotide sequence in diguanylate cyclase (*dgcE*) reveals a deletion (1456delC) **(C)** which results in a frameshift mutation at amino acid residue 486 in the protein sequence (H486fs) and a STOP codon at position 490 **(D)**. Multiple sequence alignment was performed using ClustalW in BioEdit v7.2.5).

Computational analysis of whole-genome sequence data for genes with known roles in curli production (RpoS, phosphodiesterase PdeR, diguanylate cyclase DgcM, DNA binding transcriptional activator MlrA, transcriptional dual regulator CsgD, minor curli subunit CsgB, major curli subunit CsgA, DNA-Binding protein Dps, chaperone protein DnaK, transcriptional regulator OmpR, catabolite repressor/activator protein Cra, small regulatory RNA RydC, small regulatory RNA RprA, and the *csgD* promoter region preceding *csgB*) was used to identify alleles that might impact curli production in the three curli-negative soil-persistent *E. coli* strains. RpoS was 100% conserved in the curli-positive (COB583 and COB585) as well as curli-negative *E. coli* (COB584, COB586 and COB587) while both the *csgB* and the *csgD* promoter sequences were 100% identical in them (data not shown). There were amino acid substitutions in MlrA in *E. coli* COB585 but none in the three curli-negative or the other curli-positive strains (COB583, SE11, SE15 and BW25113) compared to the reference sequence (*E. coli* K-12 W3110) (data not shown). Although soil-persistent *E. coli* COB585 and commensal strain SE15 had some amino acid substitutions in many of these regulators of curli production when compared to the reference strain, they both retained the ability to produce curli. The same amino acid substitution, Threonine (T) to Alanine (A) at codon 98 (T98A), was found in DgcM in the three curli-negative strains, relative to the reference sequence. However, other soil-persistent *E. coli* with the same allele (T98A) in *dgcM* were curli-positive (data not shown). Strikingly, analysis of the phosphodiesterase PdeR (formerly named YciR) coding sequence, regarded as the “trigger switch” for *E. coli* biofilm (Lindenberg et al., 2013), revealed a point mutation at nucleotide 1858 (1858G > A) (**Figure 3A**), which resulted in an amino acid change from Glutamate (E) to Lysine (K) at codon 620 (E620K) in the three curli-negative strains (**Figure 3B**). The mutation (E620K) occurred in the EAL domain of the phosphodiesterase PdeR, which is important for the catalytic activity of PdeR. Multiple sequence alignment of the gene encoding the cyclic-di-GMP diguanylate cyclase (*dgcE*) revealed a single base deletion (1456delC) in the three curli-negative strains (**Figure 3C**) that led to a frameshift mutation in the amino acid sequence of DgcE (H486fs), resulting in a stop codon at codon 490 (**Figure 3D**). These mutations in *pdeR* and *dgcE* were found uniquely in the three curli-negative soil-persistent *E. coli* strains thus suggesting a causative role in the curli-deficient phenotype.

**FIGURE 4:**
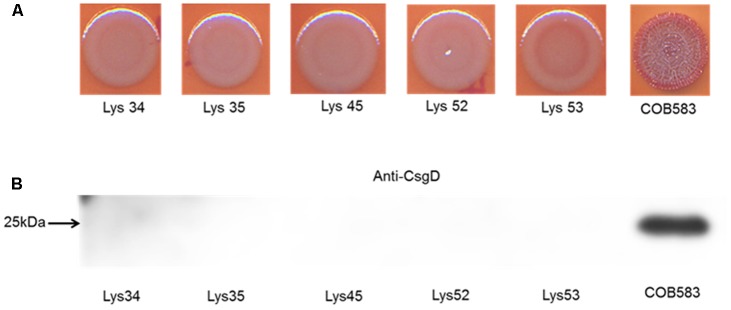
Five additional soil-persistent *Escherichia coli* with mutations in *dgcE* and *pdeR* were curli-negative. **(A)** Macrocolonies of additional soil-persistent *E. coli* with the same mutations in the nucleotide sequences of diguanylate cyclase (*dgcE*) and phosphodiesterase (*pdeR*) (*E. coli* Lys34, Lys35, Lys45, Lys52, Lys53) with positive control strain (COB583) were grown on Congo Red-containing Yeast Extract and Casamino acid (CR-YESCA) agar at 28°C for 48 h. **(B)** Western blotting was done for determining the expression of CsgD under the same conditions described above.

**FIGURE 5:**
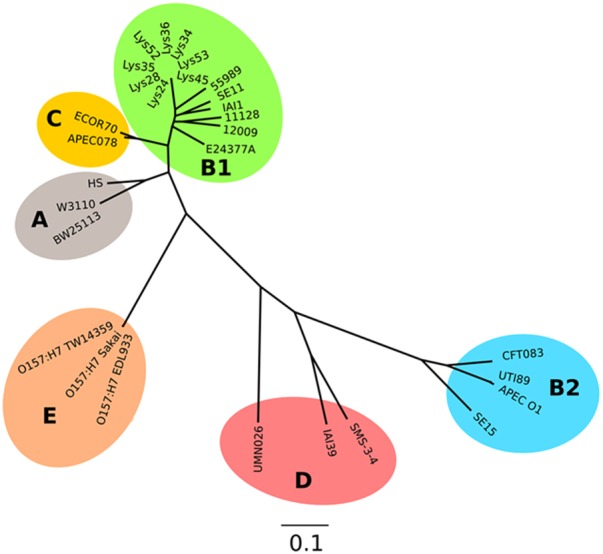
Parsimony tree of whole-genome SNPs shows relatedness of the curli-negative strains with other known *E. coli* strains. Phylogeny generated with kSNP3 using sequence data indicated in Supplementary Table S2. Non-curli producing strains form a distinct cluster within the B1 phylogroup.

Analysis of the sequences of the remaining 165 soil-persistent *E. coli* in the collection, which were obtained from different soil lysimeters, showed that the same *dgcE* and *pdeR* mutations were found in five additional soil-persistent *E. coli* strains. Interestingly, these five additional strains (Lys 34, 35, 45, 52, and 53) neither expressed CsgD nor produced curli (**Figure 4**), thus confirming that these mutations in *dgcE* and *pdeR* are associated with the loss of curli production at least on this growth medium. These eight curli-negative soil-persistent *E. coli* strains belonged to phylogenetic group B1 and clustered closely, although not all soil-persistent *E. coli* strains belonging to phylogenetic group B1 were curli-negative (**Figure 5**). Scanning Electron Microscopy (SEM) of the cells grown under the same conditions as previous experiments showed rough and wrinkled surfaces on the colonies of COB583, COB585, and BW25113, which correlates with curli production in those strains (data not shown). This wrinkled surface was absent in the curli-negative soil-persistent strains and the BW25113Δ*csgA* used as negative control.

### Complementation of *dgcE* Restores Curli Production in Curli-Negative Soil Isolates

**FIGURE 6:**
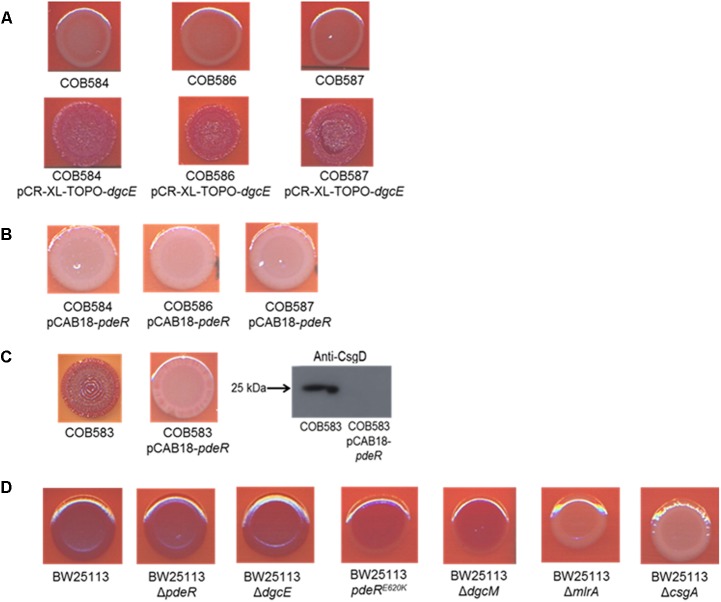
Complementation of curli-negative soil-persistent *Escherichia coli* with **(A)** wildtype DgcE restored curli production, and **(B)** PdeR had no effect on curli production. Curli-negative *E. coli* were transformed with pCR-XL-TOPO plasmid carrying wildtype *dgcE*, and pCAB18 plasmid carrying wildtype *pdeR*, grown on Congo Red-containing Yeast Extract and Casamino acid agar (CR-YESCA) at 28°C for 48 h. **(C)** Curli production in curli-positive soil-persistent *E. coli* with wildtype *pdeR* reduced curli production and CsgD protein. **(D)** Curli production among BW25113 deletion mutants grown on CR-YESCA at 28°C for 48 h.

To determine the relative importance of the two mutations identified on curli expression we endeavored to separate the alleles into separate strains. Attempts to cross wild type alleles of *dgcE* and *pdeR* into the soil strains proved technically difficult because of the low transformation efficiencies. Instead, we complemented the curli-deficient soil strains with plasmids carrying wild type copies of these two genes. The *dgcE* gene *in trans* fully restored the curli-expressing rdar morphotype on CR-YESCA medium (**Figure 6A**). In contrast, a wild type copy of *pdeR* had no significant effect on the curli expression in the curli-deficient strains (COB58, 586, and 587) (**Figure 6B**). This result was not unexpected as this phosphodiesterase has a negative influence on the expression of curli through its inhibition of MlrA (Lindenberg et al., 2013). Indeed, the negative effect of the *pdeR* gene on curli expression was clearly seen in COB583 and COB585, where it repressed curli expression (**Figure 6C** and Supplementary Figure S1).

In an attempt to understand the contribution of the PdeR^E620K^ mutation to the curli-negative phenotype of the soil strains, this allele was introduced to the K-12 strain BW25113 by allelic exchange, selecting for any recombinants that displayed reduced Congo Red staining. One BW25113 transformant was identified that had a slight reduction in CR staining and was subsequently confirmed by sequence analysis to carry the PdeR^E620K^ allele (**Figure 6D**). Hence, this allele appears to confer an intermediate curli expression phenotype, at least in this genetic background. Taken together these results suggest the frameshift mutation in *dgcE* (DgcE^H486fs^) had the dominant effect on the loss of curli in the soil-persistent strains, with a lesser contribution from the PdeR^E620K^ allele.

### Curli Enhances the Attachment of *E. coli* to Quartz Sand but Are Not Required for Long-Term Persistence in Soil

**FIGURE 7:**
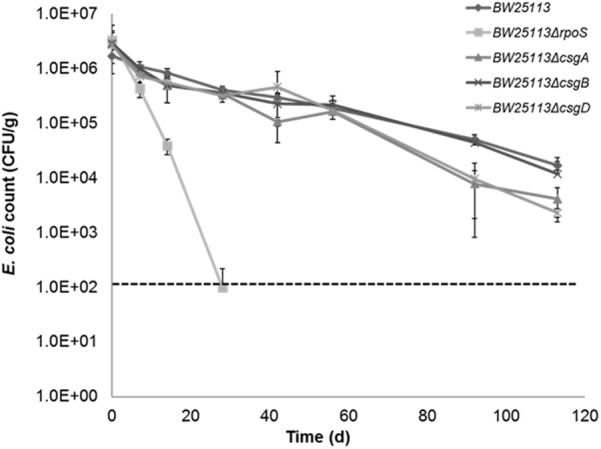
Curli are not required for *Escherichia coli* to survive in soil. Soil survival was performed by inoculating wildtype and mutants into live soil and incubated at 15°C. Dashed line represents detection limit of soil survival assay.

Although our previous study showed that there was no difference in survival between curli-positive or negative strains (Somorin et al., 2016), the contribution of curli to soil survival was analyzed in *E. coli* BW25113 using mutants with deletion in genes required for curli production. It was observed that deletion of curli subunit genes did not significantly impair soil survival in the initial 50 days in soil as seen in BW25113Δ*rpoS* (**Figure 7**). There was a small but significant defect in the survival of BW25113Δ*csgA* (*p* = 0.0084, Student’s *t*-test) and BW25113Δ*csgD* (*p* = 0.0012, Student’s *t*-test) after 113 days in soil but BW25113Δ*csgB* was not significantly different (*p* > 0.05; Student’s *t*-test) from the wild type BW25113. Since CsgB is essential for curli formation in *E. coli* (Barnhart and Chapman, 2006), this result suggested that curli are not required for long-term soil survival in this genetic background. Although the differences observed between the wildtype BW25113 and its corresponding Δ*csgA* and Δ*csgD* mutants were statistically significant at two time points (92 days and 113 days), these differences were very small and not likely to be biologically meaningful.

**FIGURE 8:**
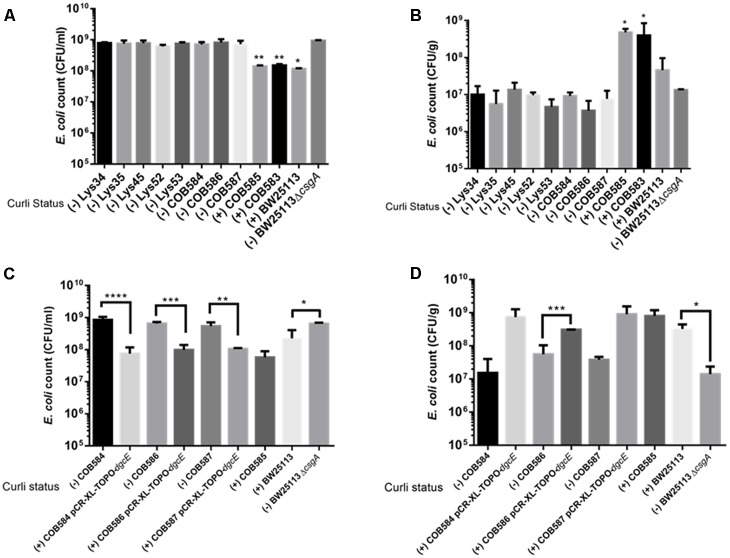
Curli-positive *Escherichia coli* attach better to quartz sand than curli-negative *E. coli.* Attachment of *E. coli* strains to quartz sand was determined after 28°C at 48 h. Planktonic cell counts **(A,C)** and biofilm cell counts **(B,D)** were determined under same condition for the wildtype and for *dgcE*-complemented curli-negative strains. Multiple comparison of the means using one-way ANOVA shows significant differences (represented by asterisks) between the curli-positive and curli-negative strains. ^∗^*p* < 0.05; ^∗∗^*p* < 0.01; ^∗∗∗^*p* < 0.001; ^∗∗∗∗^*p* < 0.0001.

Since curli enhances biofilm production in soil-persistent *E. coli* in microtiter plates, we investigated whether curli were important for attachment to an environmentally relevant surface using quartz sand. All curli-negative *E. coli* strains had significantly (*p* < 0.05, one-way ANOVA) higher planktonic cell counts than curli-positive *E. coli* indicating that fewer cells attached to the sand (**Figure 8A**). Conversely, in the same experimental setup, curli-positive strains were significantly (*p* < 0.05, one-way ANOVA) more attached to quartz sand than BW25113 Δ*csgA* and other curli-negative strains (**Figure 8B**). Attachment for the curli-positive strains was between 10- and 100-fold greater than for the curli-negative strains. There was no significant difference between the attachment of the curli negative strains (*p* > 0.05; one-way ANOVA). Complementation of curli-negative soil-persistent *E coli* with wild type DgcE, which restored curli production ability, significantly reduced planktonic cell counts and increased adhesion to quartz sand (**Figures 8C,D**). Together these data indicate that curli promote attaching to quartz sand and show that all the curli-defective soil-persistent *E. coli* strains identified in this study display reduced attachment.

## Discussion

Many studies have described curli fimbriae as being important for *E. coli* to attach to biotic and abiotic surfaces such as glass, stainless steel, and polystyrene (Cookson et al., 2002; Uhlich et al., 2009; Carter et al., 2016), but little is known about the role of curli production for soil persistence. Previously, we showed that soil-persistent *E. coli* strains varied in their ability to produce biofilm in a 96-well micro-titer plate assay (Somorin et al., 2016). In this study, we established that impaired biofilm formation was associated with a lack of curli production, and subsequently investigated the role of curli and attachment in soil survival.

Production of curli correlated with a higher level of biofilm production (Somorin et al., 2016) and attachment to a component of many soils (quartz sand) (**Figure 8**). Curli production has been shown to contribute to survival in manure-amended soil (Truhlar et al., 2015). Our data show that loss of biofilm/attachment does not affect long-term soil persistence, suggesting that some *E. coli* may occupy non-biofilm niches in the soil. The heterogeneity in curli production may reflect the genetic diversity of *E. coli* lineages present in the soil, perhaps suggesting that they have evolved to occupy different localized niches in the soil, with some of the niches not requiring the ability to retain curli. Evidence supporting this hypothesis comes from a study by Truhlar et al. (2015), which showed that the population of *E. coli* curli producers was maintained at a higher level when manure was spread on the surface of the soil than when it was injected into the soil. The selection for retention of curli on the soil surface was proposed to be based on a need for protective biofilm to help overcome UV radiation and desiccation, which would not be encountered below the soil surface (Truhlar et al., 2015).

Curli production by *E. coli* and *Salmonella* promotes macrocolony formation, community behavior and colonization of host plant and animal tissues (Gophna et al., 2001; Pande et al., 2016). Curli enhanced the attachment of *E. coli* O157:H7 to plants and stainless steel whereas mutants not producing curli showed reduced colonization of these surfaces (Carter et al., 2016). Protection against toxic metals that may be present in soil, such as mercury, is an additional benefit curli confers on *E. coli* in the environment (Hidalgo et al., 2010). Although it was hypothesized that biofilm production would increase survival of *E. coli* in the soil environment, we tested five soil-persistent *E. coli* with unknown curli status (as at then) and found that biofilm production did not confer increased soil survival (Somorin et al., 2016). This suggests that biofilm production does not provide any advantage for survival in soil under the conditions we tested. In this study, two curli-deficient mutants (BW25113Δ*csgD* and BW25113Δ*csgA*) showed a small decrease in survival compared to the wild type BW25113 at the last time two points of the assay, whereas the curli-deficient mutant (BW25113Δ*csgB*) showed no significant difference in survival compared to the wild type, suggesting soil survival is independent of the ability to produce curli (**Figure 7**). *E. coli* generally has an intrinsic ability to survive for long periods outside the host despite being thought to be primarily a gastrointestinal commensal (Ishii et al., 2006; Chiang et al., 2011; Byappanahalli et al., 2012). The finding that soil survival is independent of curli production agrees with our earlier observation that curli-positive soil-persistent *E. coli* showed no significant increase in survival compared to curli-negative soil-persistent strains (Somorin et al., 2016). The perception that persistence in the soil might depend on attachment to soil particles is challenged by our findings, thereby giving an important new insight into the lifestyle of *E. coli* outside the host.

It is possible to speculate that the soil may be exerting some selective pressures on the regulatory networks of curli production, leading to the loss of curli biosynthesis. Indeed Ravva et al. (2014) showed that exposure of *E. coli* O157:H7 to soil increased the curli-deficient subpopulation recovered from the soil. This could be because curli production is not strictly needed for long-term survival in the soil as curli-negative strains are still able to survive long-term in the soil (Somorin et al., 2016). The poor attachment of the curli-negative strains to sand (**Figure 8B**) suggests that loss of curli may also reduce biofilm formation on soil particles and could act as a strategy for environmental dissemination, allowing *E. coli* to colonize new environments and potentially new hosts. Previous studies have described that *E. coli* biofilm usually contains two subpopulations, matrix-encased and non-matrix-encased *E. coli* cells; where the non-matrix-encased cells produce no curli and are more susceptible to stress (DePas et al., 2013). The non-matrix-encased cells, which are flagellated, are considered the main agents of biofilm dispersal, giving rise to the idea that the loss of curli production observed in some of the soil-persistent *E. coli* strains may have evolved to maintain an easily dispersible population.

Although the majority of the soil-persistent *E. coli* produced curli, 10% of the strains were curli-deficient under the conditions tested. The loss of curli in the initial three curli-negative strains tested (COB584, COB586 and COB587) was not attributable to the loss of RpoS, since Western blotting showed that RpoS is expressed and functional in all of them (**Figure 2**; Somorin et al., 2016). It has previously been reported that prophage insertions into transcriptional factor *mlrA* abolished curli and biofilm production in some *E. coli* O157:H7 isolates (Uhlich et al., 2013) and non-O157 STEC (Chen et al., 2013). Truncation of *csgB* by an insertional element IS1 has also been shown to eliminate curli production in *E. coli* O78:K80 (La Ragione et al., 1999). These previously mentioned mutations were not observed in this present study. Bioinformatic analyses of some of the main genes required for curli production (such as *csgD*, *csgB*, *csgA* and *csgD* promoters) revealed wild type alleles in the strains lacking curli production. The presence of wild type curli subunit genes in *E. coli* strains do not always result in curli production and this has been reported by several authors in *E. coli* (Dyer et al., 2007; Truhlar et al., 2015), *Salmonella* spp. (De Oliveira et al., 2014) and *Enterobacter sakazakii* (Zogaj et al., 2003).

PdeR and DgcE have been identified as key regulators of curli biosynthesis (Pesavento et al., 2008; Lindenberg et al., 2013) and these two mutations (PdeR^E620K^ and DgcE^H486fs^) correlated with the inability to produce curli (and the rdar colony morphology) in COB584, COB586 and COB587 (**Figures 1**, **3**). The same mutations were found in five additional soil-persistent strains (Lys34, Lys35, Lys45, Lys52, Lys53) (**Figure 3**). All eight of these curli-negative strains belong to phylogenetic group (B1) and were isolated from two distinct lysimeters containing Rathangan soil (COB584, COB586, COB587, Lys34, Lys35, Lys45 were from Lysimeter 12; Lys52 and Lys53 were from Lysimeter 19). These two mutations are very unlikely to arise independently in separate strains in different lysimeters. The ancestor of these strains may have been introduced to the soil (or arose in the soil) over a decade or more ago. These eight strains were unable to express CsgD and hence unable to produce curli (**Figures 1**, **4**). The conserved signature GGDEF motif, which is required for diguanylate cyclase (DGC) activity and therefore cyclic-di-GMP (c-di-GMP) synthesis, is disrupted by the frameshift mutation in DgcE in the eight PdeR^E620K^ strains, which is likely to limit their capacity to synthesize c-di-GMP via DgcE, and in turn making PdeR unable to trigger curli and biofilm production in them (Lindenberg et al., 2013). The fact that the *dgcE* frameshift mutation could be fully complemented *in tran*s in the soil strains, restoring curli production (**Figure 6**), suggests that this mutation produces the dominant effect. Unsurprisingly the presence of a plasmid expressing wild type PdeR did not restore curli production in the curli-deficient soil strains as this phosphodiesterase acts negatively in modulating the activity of the MlrA transcriptional regulator (Lindenberg et al., 2013). Indeed, this plasmid repressed curli production in COB583 and COB585, curli-expressing soil persistent strains. Considering these data, it seems possible that the *pdeR* mutation (E620K) arose first in the ancestor of these curli-deficient soil strains, producing a reduced curli phenotype and that subsequently curli production was lost entirely when the *dgcE* frameshift mutation was acquired.

**FIGURE 9:**
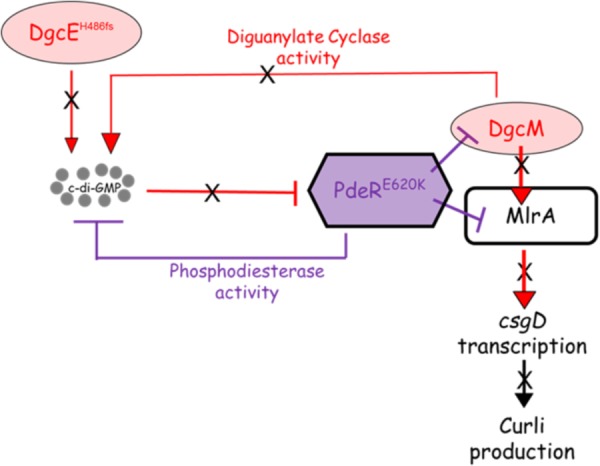
Proposed model for curli inhibition in some of the soil-persistent *Escherichia coli*. The mutated diguanylate cyclase DgcE (H486fs) in the curli-negative strains is predicted to be unable to produce sufficient cyclic di-GMP (c-di-GMP) to relieve the inhibition of MlrA-DgcM by mutated phosphodiesterase PdeR (E620K). In this state, the mutated PdeR is not able to sense c-di-GMP and thus retains the inhibition of DgcM and MlrA. This renders DgcM unable to act as transcriptional regulator to interact with MlrA and as a DGC to add to the local c-di-GMP pool, thereby inhibiting *csgD* transcription and curli production.

The proposed model explaining the basis for the loss of curli in the eight strains bearing the DgcE^H486fs^ and PdeR^E620K^ mutations is summarized in **Figure 9**. This model, which is based on the current model describing regulation of curli production in *E. coli* (Lindenberg et al., 2013), seeks to explain the loss of CsgD expression (**Figures 2**, **4**), which in turn prevents the transcription of *csgBAC* and thereby blocks curli production. In wild type cells, c-di-GMP binds PdeR causing it to dissociate from the PdeR-MlrA-DgcM complex, which in turn allows DgcM to form productive interactions with MlrA, stimulating its activity as a transcriptional regulator and as a DGC to produce more local c-di-GMP to further prevent the inhibitory activity of PdeR. The *dgcE* mutation is predicted to result in a drop in the local c-di-GMP pool, which is insufficient to dissociate PdeR from the PdeR-MlrA-DgcM complex. The *pdeR* mutation probably affects the affinity of PdeR for c-di-GMP (because the E620K change results in a charge change in a region that is very close to the active site) and this exacerbates the effect of the reduced pool of c-di-GMP. This is suggested by the reduction in CR binding that was observed when the E620K mutation was introduced into *pdeR* in BW25113. This model will need to be tested further to fully understand the mechanism behind the curli inhibition in these strains. It will be important to measure c-di-GMP levels in these curli-negative strains to determine if reduced c-di-GMP level correlates with the curli-negative phenotype. Secondly, it will be important to establish the effect of the E620K change in PdeR on the binding and hydrolysis of c-di-GMP. The interaction of the mutated PdeR with DgcM and MlrA from these strains should also be investigated, which would likely provide further insights into the regulation of the trigger mechanism.

Analysis of the EAL domains from different bacterial species showed that active EALs have glutamate (E) at codon 620 (E620) (Supplementary Figure S2). This suggests that E620 may play some role in the catalytic activity of PDEs, in addition to the previously identified conserved glutamate residue (equivalent to glutamate (E) at codon 617 in PdeR) in other functional phosphodiesterases (Rao et al., 2008). PdeR^E620K^ may be unable to play its role in c-di-GMP metabolism since the E620 of PdeR is possibly involved in its catalytic activity. Tchigvintsev et al. (2010) showed that the second guanine base of c-di-GMP interacts electrostatically with conserved glutamate at codon 706 (E706) in TBD1265 of *Thiobacillus denitrificans* (equivalent to E620 in PdeR of *E. coli*). Rao et al. (2008) observed that although mutation of E355 in RocR of *P. aeruginosa* (equivalent to E620 in *E. coli* PdeR) plays a minor role in catalysis of c-di-GMP, the distal location of the residue makes it likely to play an important role in maintaining the conformational structure required for c-di-GMP binding. Amino acid residues distal to active site residues have been shown to play crucial roles in enhancing the catalytic activity of enzymes through structure stabilization (Rajagopalan et al., 2002; He et al., 2015). Based on this, PdeR^E620K^ may cause structural changes making it difficult for the conserved residue E617 to be catalytically active. This assumption becomes important since the eight curli-negative strains in this study retained all the conserved residues previously reported to be important for PDE activity of PdeR (Römling et al., 2013). E620 is conserved in all *E. coli* PdeR searched in the National Center for Biotechnology Information (NCBI) database and no strain had K620 in its PdeR (data not shown). This tight conservation of E620 in EAL domains of different bacteria suggests a possible role for them in regulating curli production.

## Conclusion

This study identified a significant number of soil dwelling *E. coli* strains that do not produce either curli or biofilm and have a defect in attachment to quartz sand, yet are able to reside in this habitat for long periods of time. This shows that inability to produce biofilm does not compromise the ability of *E. coli* to inhabit a soil environment. For eight of the curli-defective strains identified in this study, the loss of curli was attributed to a defect in c-di-GMP signaling that leads to a failure to express the curli regulator CsgD. The data also highlight residue 620 of the phosphodiesterase PdeR as being critical for its normal activity. Finally, the results suggest that dissemination of *E. coli* in the environment could be facilitated by the loss of curli production.

## Author Contributions

YS and CO conceived the study. YS performed Congo Red Assay, western blotting, genomic comparison, SEM, soil survival, and statistical analysis. TV performed sand attachment assay and constructed mutants. NW and LP performed phylogenetic analysis. FA and FB provided the soil-persistent *E. coli* isolates used in the study. YS, TV, and CO wrote the manuscript, with contributions from NW, LP, FA, and FB. All authors reviewed the manuscript and approved the final draft of the manuscript.

## Conflict of Interest Statement

The authors declare that the research was conducted in the absence of any commercial or financial relationships that could be construed as a potential conflict of interest.

## Footnotes

1 https://ecocyc.org/
