# Supplementary material for: Absence of Curli in Soil-Persistent Escherichia coli Is Mediated by a C-di-GMP Signaling Defect and Suggests Evidence of Biofilm-Independent Niche Specialization
Source: Front Microbiol. 2018 Jun 22;9:1340. doi: 10.3389/fmicb.2018.01340 (PMC6029578; doi:10.3389/fmicb.2018.01340)
Supplement: Supplementary file 1 [file Data_Sheet_1.pdf]

Table S1: List of strains used in the study

Strains	Phylogenetic Group	Habitat	Source
COB583 (Lys9)	C	Soil	F. Brennan (Teagasc, Ireland)
COB583 Δ*rpoS*	-	-	This study
COB584 (Lys24)	B1	Soil	This study
COB585 (Lys25)	E	Soil	This study
COB586 (Lys28)	B1	Soil	This study
COB587 (Lys36)	B1	Soil	This study
SE11	B1	Commensal	RIKEN BRC, Japan
SE15	B2	Commensal	RIKEN BRC, Japan
BW25113	A	Lab strain	NBRP (Japan)
BW25113 Δ*rpoS*	-	-	NBRP (Japan)
BW25113 Δ*csgA*	-	-	NBRP (Japan)
BW25113 Δ*csgB*	-	-	NBRP (Japan)
BW25113 Δ*csgD*	-	-	NBRP (Japan)
BW25113 Δ*bcsA*	-	-	NBRP (Japan)
Lys34	B1	Soil	F. Abram (NUIG, Ireland)
Lys35	B1	Soil	F. Abram (NUIG, Ireland)
Lys45	B1	Soil	F. Abram (NUIG, Ireland)
Lys52	B1	Soil	F. Abram (NUIG, Ireland)
Lys53	B1	Soil	F. Abram (NUIG, Ireland)

Table S2: Description of genomes used for phylogenetic analysis

Accession #	Strain	Phylogenetic Group
AE014075	CFT083	B2
AP009240	SE11	B1
AP009378	SE15	B2
NC_002695	O157H7_Sakai	E
NC_007779	W3110	A
NC_007946	UTI89	B2
NC_008563	APECO1	B2
NC_009800	HS	A
NC_009801	E24377A	B1
NC_010498	SMS34	D
NC_011741	IAI1	B1
NC_011748	55989	B1
NC_011750	IAI39	D
NC_011751	UMN026	D
NC_013008	O157H7_TW14359	E
NC_013353	12009	B1
NC_013364	11128	B1
NZ_CP008957	O157H7_EDL933	E
NZ_CP009273	BW25113	A
CP004009	APEC078	C
GCF_002190075.1_ASM219007v1*	ECOR70	C

*from NCBI Assembly Database

Table S3: Description of 17 curli-negative *Escherichia coli* from soil collection

Strain ID	Phylogenetic Group	Lysimeter Source	Date Isolated
Lys15	A	19	March 2007
Lys23	A	12	September 2010
Lys24	B1	12	September 2010
Lys28	B1	12	September 2010
Lys34	B1	12	September 2010
Lys35	B1	12	September 2010
Lys36	B1	12	September 2010
Lys52	B1	19	September 2010
Lys53	B1	19	September 2010
Lys89	B1	19	August 2012
Lys56	B2	19	May 2012
Lys77	B2	43	August 2012
Lys109	B2	12	August 2012
Lys113	B2	12	August 2012
Lys45	B1	12	September 2010
Lys54	E	43	September 2010
Lys57	unknown	43	May 2012

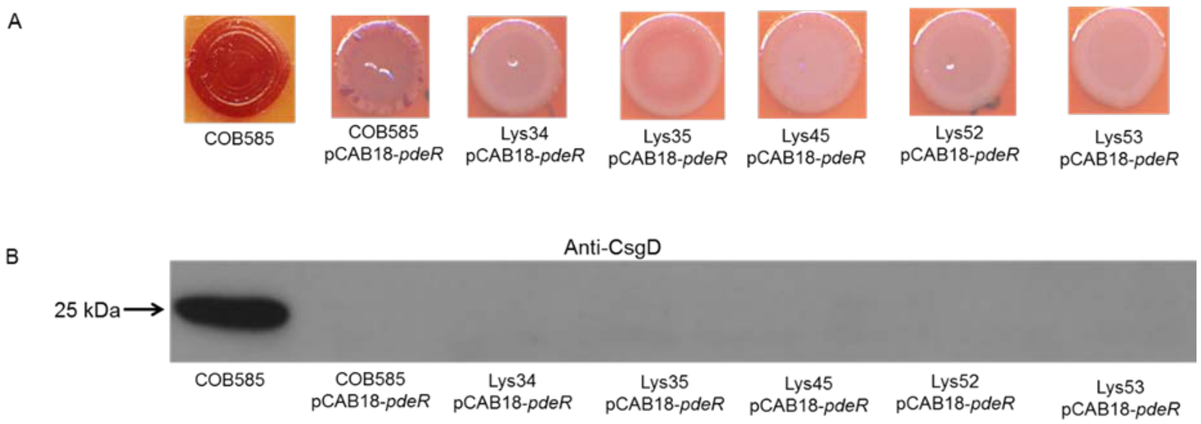

Figure S1. Complementation of curli-negative soil-persistent *Escherichia coli* with wild type PdeR did not restore curli production. (A) Curli-positive and curli-negative *E. coli* were transformed with pCAB18 plasmid carrying wild type *pdeR* and the strains were grown on Congo Red-containing Yeast Extract and Casamino acid agar (CR-YESCA) with IPTG and Ampicillin at 28°C for 48 h. (B) Western blot analyses of CsgD expression in the same set of strains but grown on YESCA agar medium without the congo red.

```
YkuI_Bs         ESSNLDRIALLSPDLLKIDLQALKVSQPSPSYEHVLYSISLLARKIGAALLYEDIEANFQ 216
PdeA_Gx         GFSSLSRLTRLPLTEIKIDRSFIMNLDHDPNAQAVTTAVIGIGNRLGMTVVTEGVETEAQ 701
TBD1265_TBD     GYSSLSYLSQLPFHGLKIDQSFVRKIPAHPSETQIVTTILALARGLGMEVVAEGIETAQQ 710
PdeR_COB584     GYSSLSQLARFPIDAIKLDQVFVRDIHKQPVSQSLVRAIVAVAQALNLQVIAEGVKSAKE 624
PdeR_W3110      GYSSLSQLARFPIDAIKLDQVFVRDIHKQPVSQSLVRAIVAVAQALNLQVIAEGVESAKE 624
RocR_PAO1       GYSSLDRLCEFPFSQIKLDRTFVQKMKTQPRSCAVISSVVALAQALGISLVVEGVESDEQ 359
VieA_VCV52      GYASLGQLAQLPFTELKIDRSFVHDLATNYKHQQLTNMCLLLAQSLGLHCVVEGVENEET 350
                    :  :  :    :*:*   :           :      : .      :   ::
```

Figure S2. Multiple Sequence Alignment of EAL domains in some bacterial species. Multiple sequence alignment of EAL domain in some bacteria showing conserved glutamate (E) at positions equivalent to codon 620 in PdeR in *E. coli* (underlined). Previously reported glutamate (E) conserved for phosphodiesterase activity, corresponding to codon 617 of PdeR in *E. coli*, is in bold. YkuI from *Bacillus subtilis* (YkuI_Bs); PdeA from *Gluconacetobacter xylinus* (PdeA_Gx); TBD1265 from *Theobacillus denitrificans* (TBD1265_TBD); PdeR from *E. coli* COB584 (PdeR_COB584); PdeR from *E. coli* W3110 (PdeR_W3110); RocR from *Pseudomonas aeruginosa* PAO1 (RocR_PAO1) and VieA from *Vibrio cholerae* VCV52 (VieA_VCV52). Multiple sequence alignment was performed using ClustalOmega.
